# Septins from the Phytopathogenic Fungus *Ustilago maydis* Are Required for Proper Morphogenesis but Dispensable for Virulence

**DOI:** 10.1371/journal.pone.0012933

**Published:** 2010-09-27

**Authors:** Isabel Alvarez-Tabarés, José Pérez-Martín

**Affiliations:** Departamento de Biotecnología Microbiana, Centro Nacional de Biotecnología CSIC, Madrid, Spain; University of Wisconsin - Madison, United States of America

## Abstract

**Background:**

Septins are a highly conserved family of GTP-binding proteins involved in multiple cellular functions, including cell division and morphogenesis. Studies of septins in fungal cells underpin a clear correlation between septin-based structures and fungal morphology, providing clues to understand the molecular frame behind the varied morphologies found in fungal world.

**Methodology/Principal Findings:**

*Ustilago maydis* genome has the ability to encode four septins. Here, using loss-of-function as well as GFP-tagged alleles of these septin genes, we investigated the roles of septins in the morphogenesis of this basidiomycete fungus. We described that septins in *U. maydis* could assemble into at least three different structures coexisting in the same cell: bud neck collars, band-like structures at the growing tip, and long septin fibers that run from pole to pole near the cell cortex. We also found that in the absence of septins, *U. maydis* cells lost their elongated shape, became wider at the central region and ended up losing their polarity, pointing to an important role of septins in the morphogenesis of this fungus. These morphological defects were alleviated in the presence of an osmotic stabilizer suggesting that absence of septins affected the proper formation of the cell wall, which was coherent with a higher sensitivity of septin defective cells to drugs that affect cell wall construction as well as exocytosis. As *U. maydis* is a phytopathogen, we analyzed the role of septins in virulence and found that in spite of the described morphological defects, septin mutants were virulent in corn plants.

**Conclusions/Significance:**

Our results indicated a major role of septins in morphogenesis in *U. maydis*. However, in contrast to studies in other fungal pathogens, in which septins were reported to be necessary during the infection process, we found a minor role of septins during corn infection by *U. maydis*.

## Introduction

Septins are a highly conserved family of guanosine triphosphate (GTP)-binding proteins that assemble into heteromeric polymers. Although they were initially described as essential elements during cytokinesis in *Saccharomyces cerevisiae* [1], their roles expanded to in a wide range of organisms from yeast to metazoan as well as in a variety of other cellular processes such as vesicular transport, organization of actin and microtubule cytoskeletons, cell division, and in various neurodegenerative diseases and cancer [2]. Septins seem to perform two main non-catalytic functions: septin-based structures that are closely associated with membranes provide a boundary that restricts certain determinants to particular cortical domains; In addition, septin-based structures serve as scaffolds necessary for the localization of many factors involved in polarity and cell division [3].

In fungi, septins seem to play an important role in morphogenesis. In agreement to the heterogeneity in shapes and sizes observed in the fungal world, septin structures in fungi are formed with strikingly different appearances and potentially different functions within a single cell [4], [5], [6]. It is thought that studies about septins in fungi could help to understand morphogenetic processes that are intrinsic part of fungal development. One of these processes refers to the ability of fungal pathogens to undergo morphological transformations during host invasion. More importantly, morphogenetic changes have been strongly implicated as virulence determinants [7], and therefore it seems obvious that septins were shown to be necessary for virulence in several pathogenic fungi [8].

The corn smut fungus, *Ustilago maydis*, is an excellent model system for the analysis of the molecular basis of fungal plant pathogenicity [9], [10]. This basidiomycete fungus belongs to an important group of plant pathogens, the smut fungi, which can cause considerable grain yield loss and economic damage. Previous research had indicated that cytoskeleton regulators, like Rac1, or molecular motors such as myosin V, play roles during pathogenic development in *U. maydis* [11], [12]. However, a cautionary note is required, as several results also underpin that in this organism fungal morphogenesis could be a component but not a complete explanation of host invasion. For instance, cells defective in some regulators of polar growth such as Spa2 or the Cdk5-associated cyclin Pcl12 were as virulent as wild-type cells in standard virulence assays [13], [14]. In the same way, an early report about a septin in *U. maydis* [15] described that in this fungus septin Sep3 seems to have a minor role in infection. As this result contrasts with recent reports highlighting a role of septins during the infection process in other fungi, including the human pathogenic fungi *Cryptococcus neoformans* and *Candida albicans* [16], [17] we decided to characterize septins in *U. maydis* in more detailed.

Genomic data mining has revealed the presence of four different septins (named Sep1–4) in *Ustilago maydis* [18]. *U. maydis* Sep3 was already described but information about its subcellular localization was not provided [15]. Following the phylogenetic tree and nomenclature proposed by Momany and colleagues [18], Sep1 belongs to Group 2A, which includes *S. cerevisiae* Cdc3p, Sep2 to Group 4, which includes *S. cerevisiae* Cdc12p, Sep3 to Group 3, which includes *S. cerevisiae* Cdc11p and Sep4 to Group 1A, which includes *S. cerevisiae* Cdc10p.

Here, we describe that septins in *U. maydis* can be assembled into different higher-order structures, one of them being long septin fibers that partially co-aligned with the microtubule cytoskeleton. Deletion of septin genes severely affects cell morphology and provokes thermosensitivity as well as enhanced sensitivity to cell wall stressors, suggesting a defective construction of the cell wall in the absence of septins in *U. maydis*. Importantly, we found that none of the septin mutants were avirulent, suggesting that these structures play a minor role during the virulence process.

## Results

### Different septin structures coexist in *U. maydis* cells

As a starting point, we decided to study the subcellular localization of septins in *U. maydis*. For this, we constructed *U. maydis* strains expressing amino-terminal GFP-tagged versions of each septin gene under their own respective promoters. These GFP-Septin strains showed similar growth rates and morphology to wild-type cells, indicating that the N-terminal tagging did not interfere with septin functions (see below for a description of the phenotype of septin mutants).

**Figure 1:**
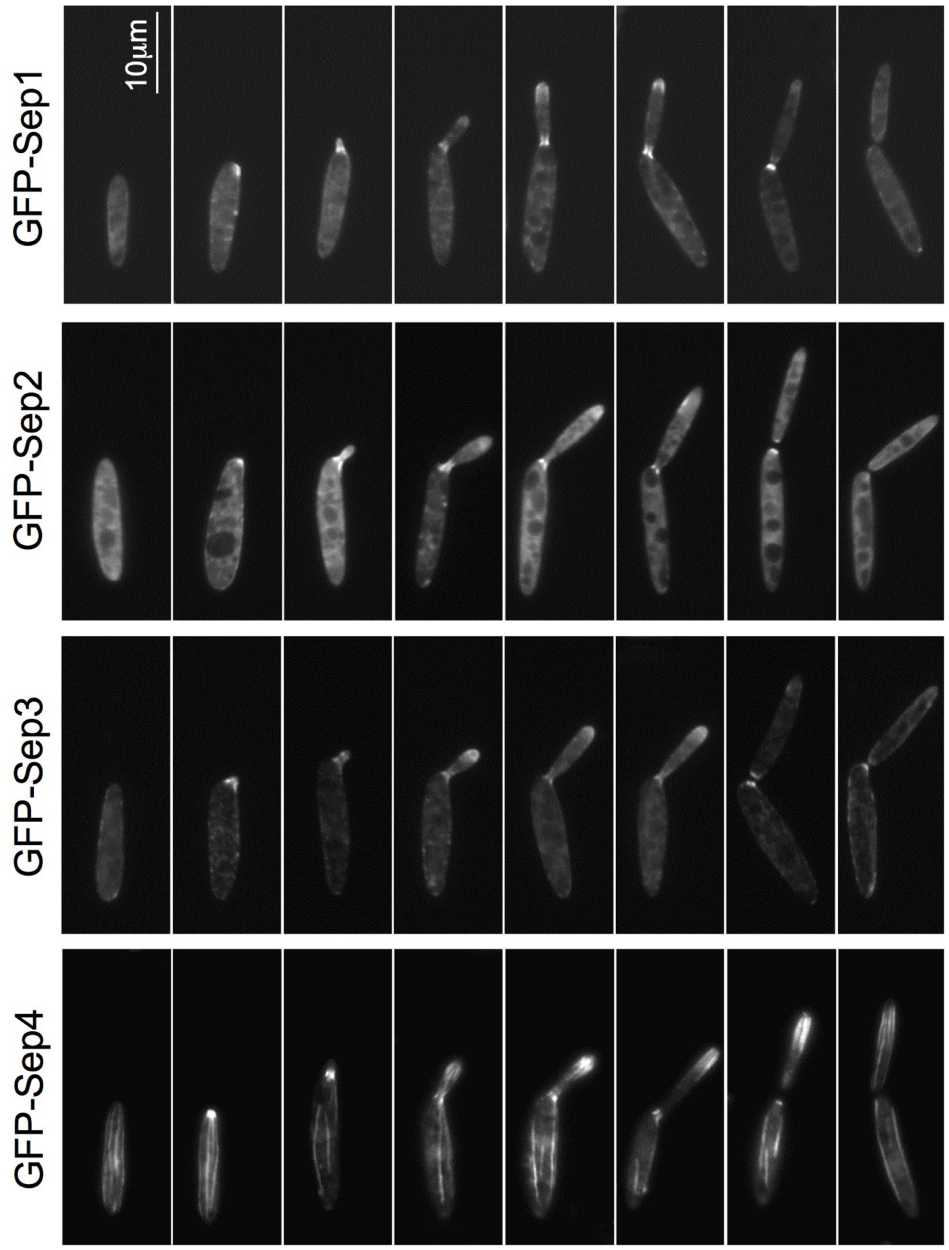
Septins localization throughout the cell cycle. Cells expressing N-terminal GFP-tagged Sep1-4 under their native loci were grown to log phase at 28°C and observed with a widefield fluorescence microscope. Representative images were chosen to show the progression of septin localization throughout the cell cycle.

**Figure 2:**
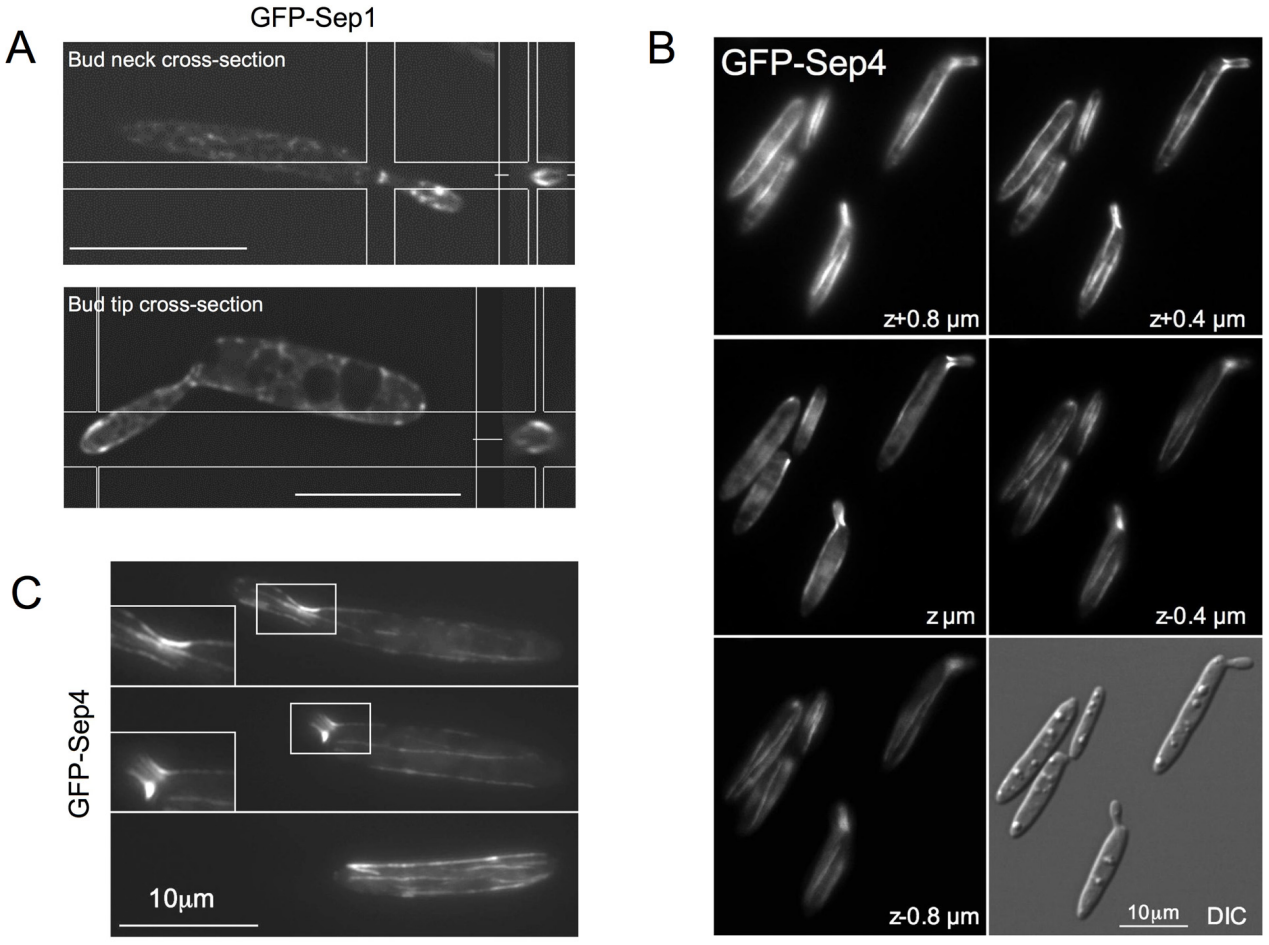
Septins subcellular localization. A. DeltaVision captured images were deconvolved and cross-section images of the bud neck and the bud tip were obtained using the Imaris 6.0.1 software. B. Images of the complete volume of GFP-Sep4 cells were taken every 0.2 µm. A selection of images was chosen to show that the filaments run close to the cortex but no throughout the middle of the cytoplasm (middle section, z). C. GFP-Sep4 cells were grown to log phase and observed in a DeltaVision microscope. Representative images of different cell cycle stages are shown. Insets show a zoom in of the bud neck in which the filaments crossing through can be observed.

We found that the four septins localized at the bud neck in a similar way to what it has been previously shown in other budding yeasts such as *S. cerevisiae* or *C. albicans*; a structure appeared at the future bud site just before bud emergence and it remained at the bud neck during the budding process [19]–[22] (Fig. 1). A cross-section of the neck showed that septins in *U. maydis* form a collar-like structure (Fig. 2A).

We also observed that all four septins were located at the bud, forming a band-like structure just behind the tip (Fig. 1). This structure appeared to surround the cytoplasm just beneath the cortex such as in a cross-section it appeared as a circumference (Fig. 2A). In *S. cerevisiae*, septin localization to a yeast bud tip is a common effect of a variety of mutations that block assembly of the septin collar at the bud neck, what has been suggested to be a consequence of the ability of certain polarity factors to retain the capacity to recruit septins if normal collar localization is perturbed [23]–[29]. However, in our case we were able to observe in the same cell both structures, neck collar and tip band, so we consider unlikely that the apical localization of septins is a consequence of defects in the neck collar formation.

The third septin structures we observed were long septin fibers running along the major axis of the cell from pole to pole (Figs. 1 and 2B). These fibers were observed only with GFP-Sep4, suggesting either that they were composed exclusively of Sep4 or alternatively that the amount of the other septins in the fiber was below our level of detection (see below for dependencies upon other septins in fiber formation). Moreover, it remains unknown whether these fibers are just single filaments or filament bundles. We found similar structures when GFP was appended to Sep4 at the C-terminus end (not shown). However, to rule out that GFP was not producing aggregates that form fibers, we confirmed such structures by immunofluorescence using a Sep4 tagged with a small epitope. Between 3 and 6 fibers per cell could be observed, though the majority of cells had 4 fibers (Fig. S1). In contrast to the other septin structures observed at the bud neck and at the bud tip -whose localization changed over the cell cycle- Sep4 fibers were present throughout the cell cycle. In unbudded cells, fibers run from one pole to another along the long axis of the cell. These fibers appeared to run parallel and near to the cell cortex (Movie S1). In cells with small buds it was possible to observe fibers crossing the bud neck and reaching the bud (Fig. 2C). In cells with large buds, they were present in both the mother and the bud cells.

### Septins are required for cell integrity and morphogenesis

**Figure 3:**
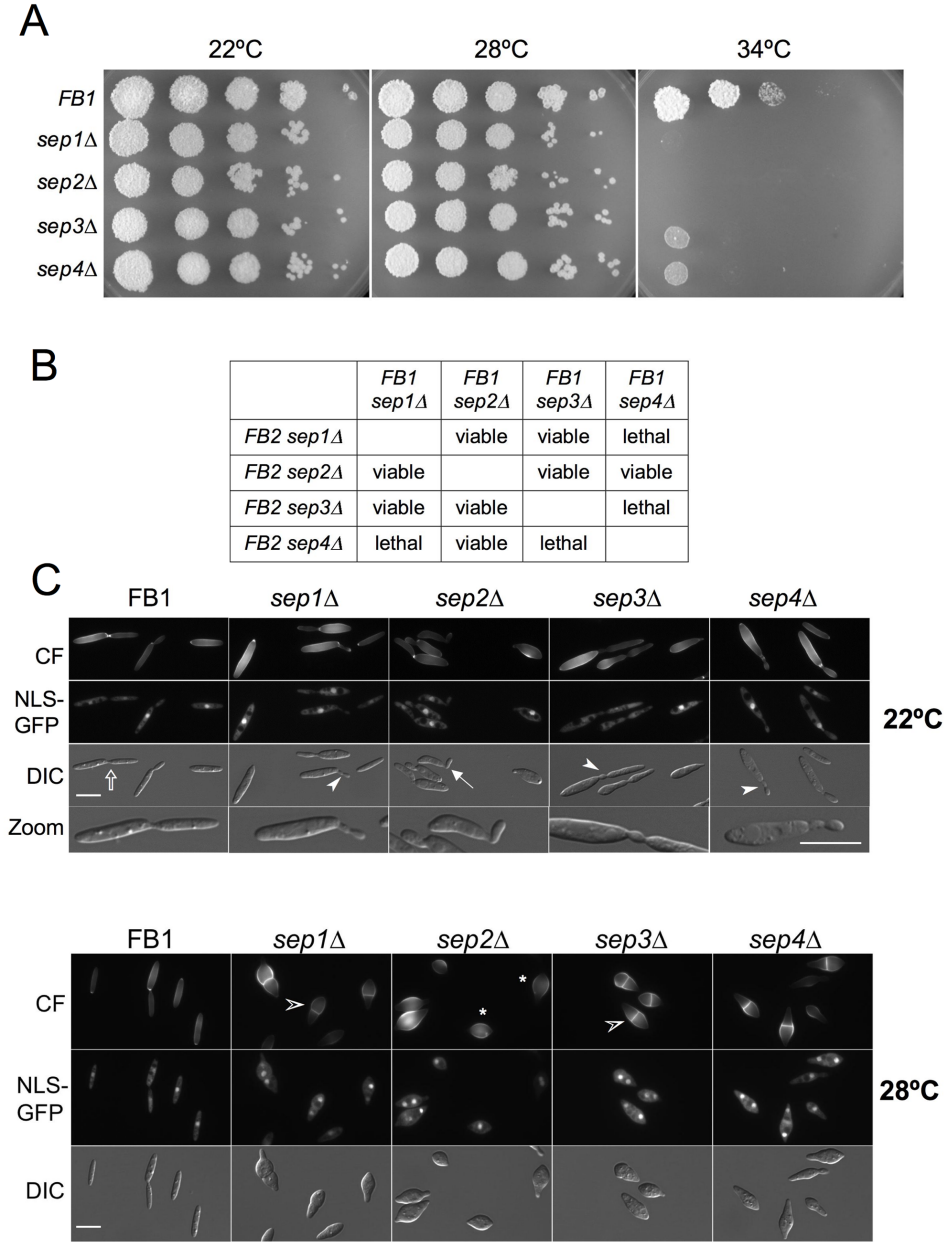
Septin deletion mutants display altered cell morphology and temperature sensitivity. A. Spot test of tenfold serial dilutions of wild-type and *sep1-4* deletion mutants grown at different temperatures. Septin deletion strains were unable to grow at 34°C. B. Double septin deletion mutants. *sep4*Δ*sep1*Δ and *sep4*Δ*sep3*Δ strains were lethal while the rest of possible combinations were viable. C. Wild-type and septin deletion mutant cells expressing a nuclear localisation signal tagged with GFP (NLS-GFP) were grown to log phase at 22°C (top panel) or 28°C (bottom panel) and stained with calcofluor (CF) to observe the cell wall. Widefield microscope images were captured. In comparison with wild-type cells, at 22°C a minor bud neck defect was observed in mutant cells (bendy (arrowhead) and wider (arrow) bud necks). However, at 28°C septin deleted cells showed a strong morphology defect characterised by a swollen region in the middle of the cell (asterisk) strongly stained by calcofluor. Cells became rounded at the centre but maintained some polar growth at their tips and divided by placing a septum across the middle of the cell (empty arrowhead).

The coexistence of multiple distinct septin structures in *U. maydis* -some of them not associated with the bud neck- suggests roles of septins further than bud neck formation. To address the function of septins in *U. maydis*, a deletion analysis was carried out. We deleted separately each of the septin genes. In contrast to what was described in *S. cerevisiae* [1], [19], [20], [21], [30] and *C. albicans* [22], none of the septins was essential. However, all single septin mutants were unable to grow in solid medium at 34°C (Fig. 3A). We also obtained double septin deletion mutants and found that only two combinations showed synthetic lethality (*sep1*Δ *sep4*Δ and *sep3*Δ *sep4*Δ) while the rest of possible combinations were viable (Fig. 3B). Triple *sep1*Δ *sep2*Δ *sep3*Δ combination was also lethal (not shown). Double mutant combinations that were viable behaved in a similar way as single mutants attending to morphology and temperature (not shown).

**Figure 4:**
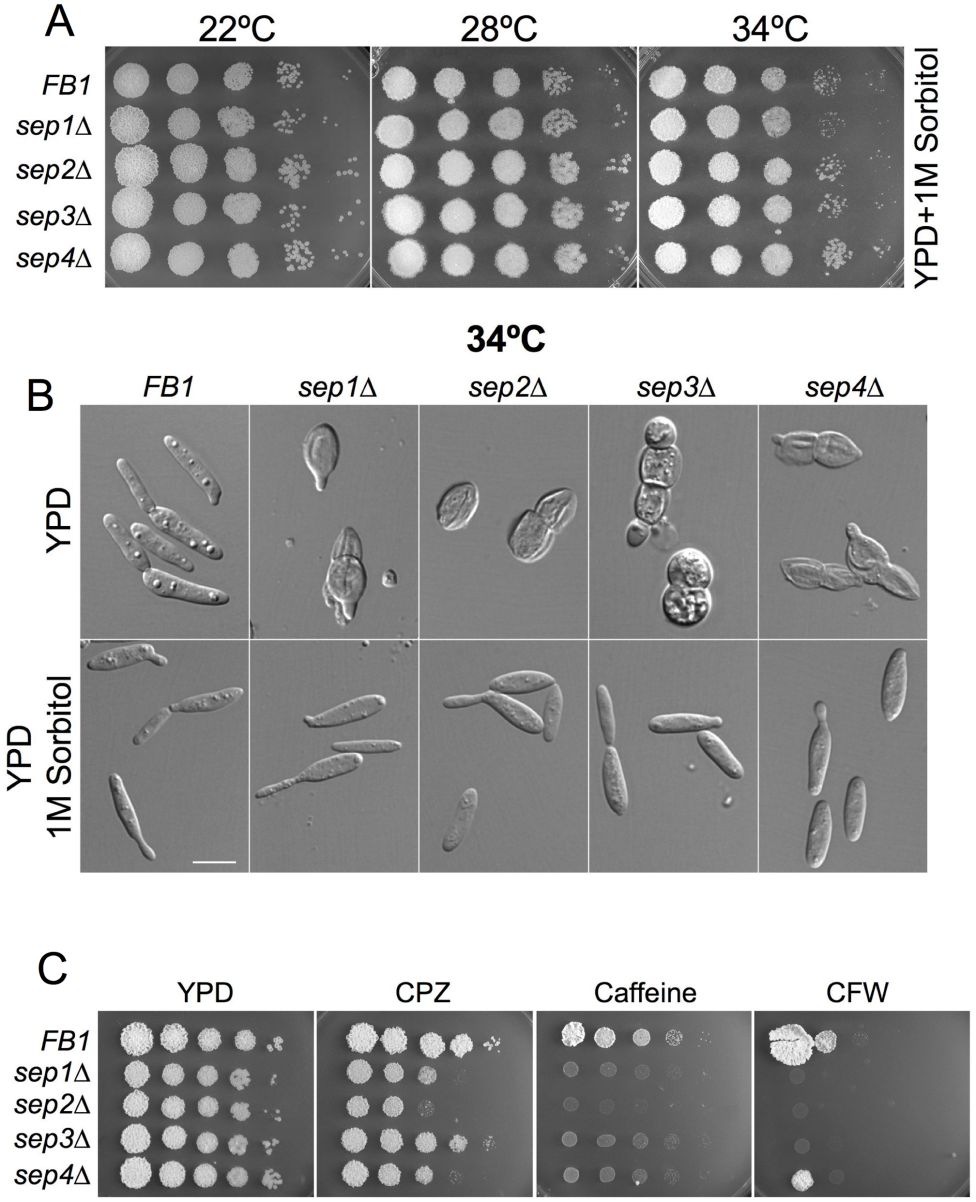
Sensitivity of septin mutants to temperature and cell wall inhibitors. Rescue of phenotypes by 1M sorbitol. A. Spot tests of tenfold serial dilutions of wild type and septin deletion mutants grown onto YPD with 1M sorbitol at different temperatures. The termosensitivity of septin mutants was rescued by the osmoregulator sorbitol. B. Wild type and septin deletion mutant cells were grown to log phase at 34°C and observed with a widefield fluorescence microscope. At 34°C septin mutant cells became rounded, placed a septum at the central region to divide and presented a cell separation defect. Finally they died by cell lysis. All these phenotypes were rescued by addition of 1M sorbitol. Bar: 10 µm**.** C. Spot test of tenfold serial dilutions of wild type and septin deletion mutants grown with the cell wall inhibitors calcofluor white (CFW), chlorpromazine (CPZ) and caffeine (Caff).

Mutant strains were analyzed microscopically at different temperatures. For this, cells were grown at permissive temperature (22°C) and then shifted to different temperatures. When grown at 22°C, cells displayed their characteristic elongated, cigar-shape morphology although frequently they were thicker than wild-type cells and showed minor bud neck defects (i.e. 32% of the cells had bendy or wider bud necks, n = 100 cells; Fig. 3C). However, when grown at 28°C, 92% of the cells (n = 100) swollen in the central region of the cell and placed a septa across the middle of the cell (Fig. 3C). At 34°C, cells showed a strong lysis phenotype as evidenced by the frequent presence of cell “ghosts” and cell debris (Fig. 4B, upper row).

We found that the temperature-sensitive growth, the cell lysis defect and the morphological defect of the mutants were rescued by 1 M sorbitol, an osmotic stabilizer, known to rescue cell wall defects, suggesting that the primary defect might be a defective cell wall structure (Fig. 4A, B). These results suggested that septins in *U. maydis* were required to maintain cell integrity. Therefore, we tested the sensitivity of septin mutant cells at permissive temperature (22°C) to compounds described to affect cell wall integrity in this fungus (i. e. caffeine, Calcofluor White and chlorpromazine) [31]. Accordingly, septin defective cells showed higher sensitivity than wild-type cells to these compounds (Fig. 4C).

**Figure 5:**
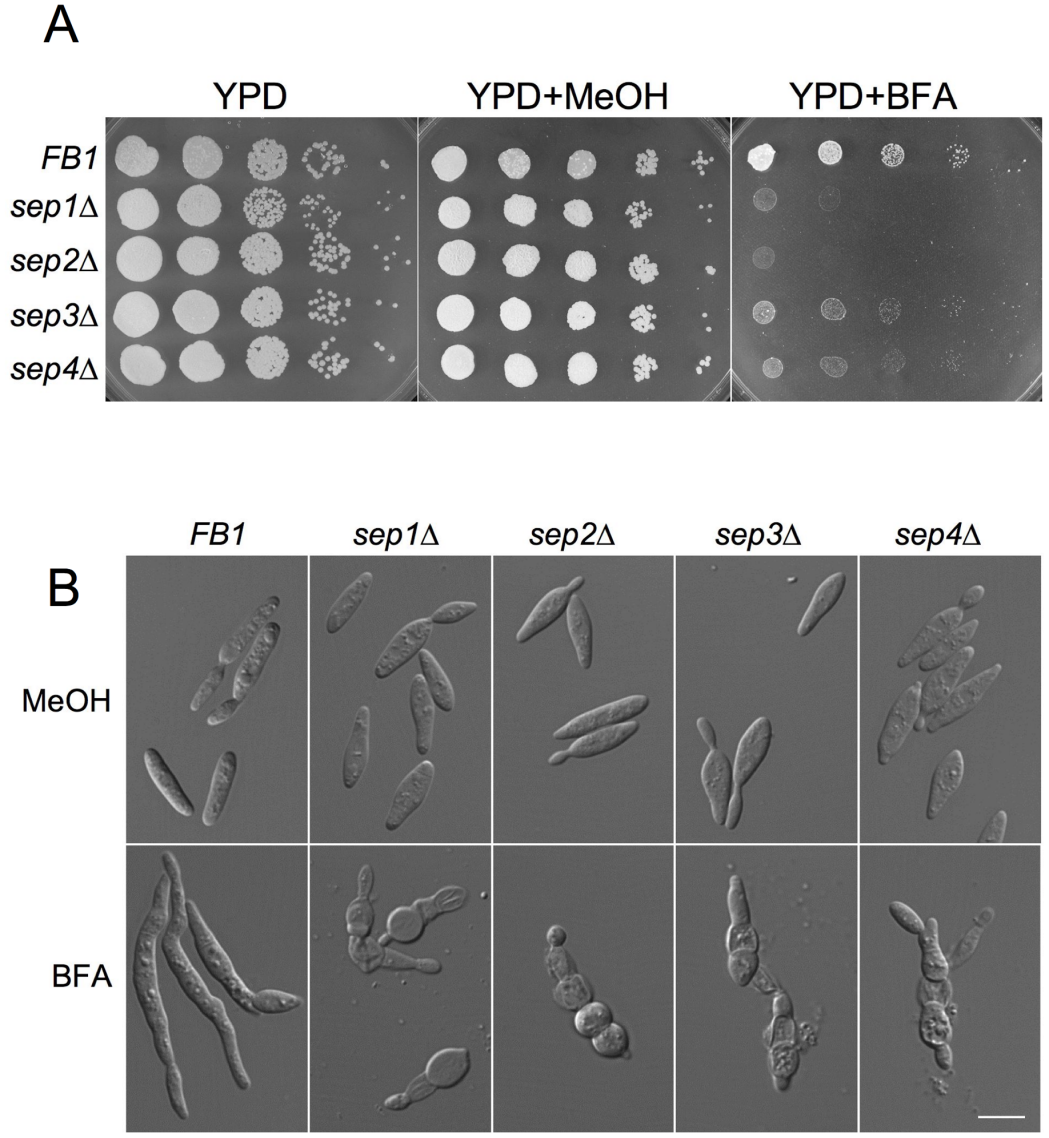
Septin deletion mutants are more sensitive to BFA. A. Spot test of tenfold serial dilutions of wild type and septin deletion mutants grown at 22°C onto YPD with brefeldin A (BFA, 25 µM) or its solvent, methanol. Septin mutants were more sensitive than wild type cells to BFA. B. Wild type and septin deletion mutants cells were grown to log phase at 22°C in YPD and treated with BFA (200 µM) or its solvent methanol for 12 h. BFA treatment mildly affected wild type cell morphology. In contrast, septin mutant cells became rounded and lysated. In both cases a cell separation delay was observed. Bar: 10 µm.

Septins were proposed to be involved in exocytosis [32] and cell wall defects can be explained, among others, by defective exocytosis. We checked whether *U. maydis* septin defective cells were more sensitive than wild-type cells to sublethal concentration of Brefeldin A (BFA) at permissive temperature (22°C). BFA has been used to block secretion in *S. cerevisiae* [33], [34] and it is active against *U. maydis* cells, perturbing apical secretion [35]. We found that septin mutant strains were more sensitive to BFA (Fig. 5A). Furthermore, when grown in the presence of BFA, we found a synthetic enhancement of the morphological defects observed in septin mutants: addition of BFA at permissive temperature (22°C) mimicked the terminal phenotype of septin mutants at high temperature (34°C): round cells that eventually lysed leaving behind cell debris and “ghosts” (Fig. 5B).

### Interdependence of septins in the distinct sub-cellular structures

To determine which septins molecules or assemblies of molecules were critical for the different localization patterns, we used the different GFP-tagged strains combined with the different deletion alleles. We grew cells at low temperature (22°C), since at 28°C no structures were observed in any combination (not shown).

**Figure 6:**
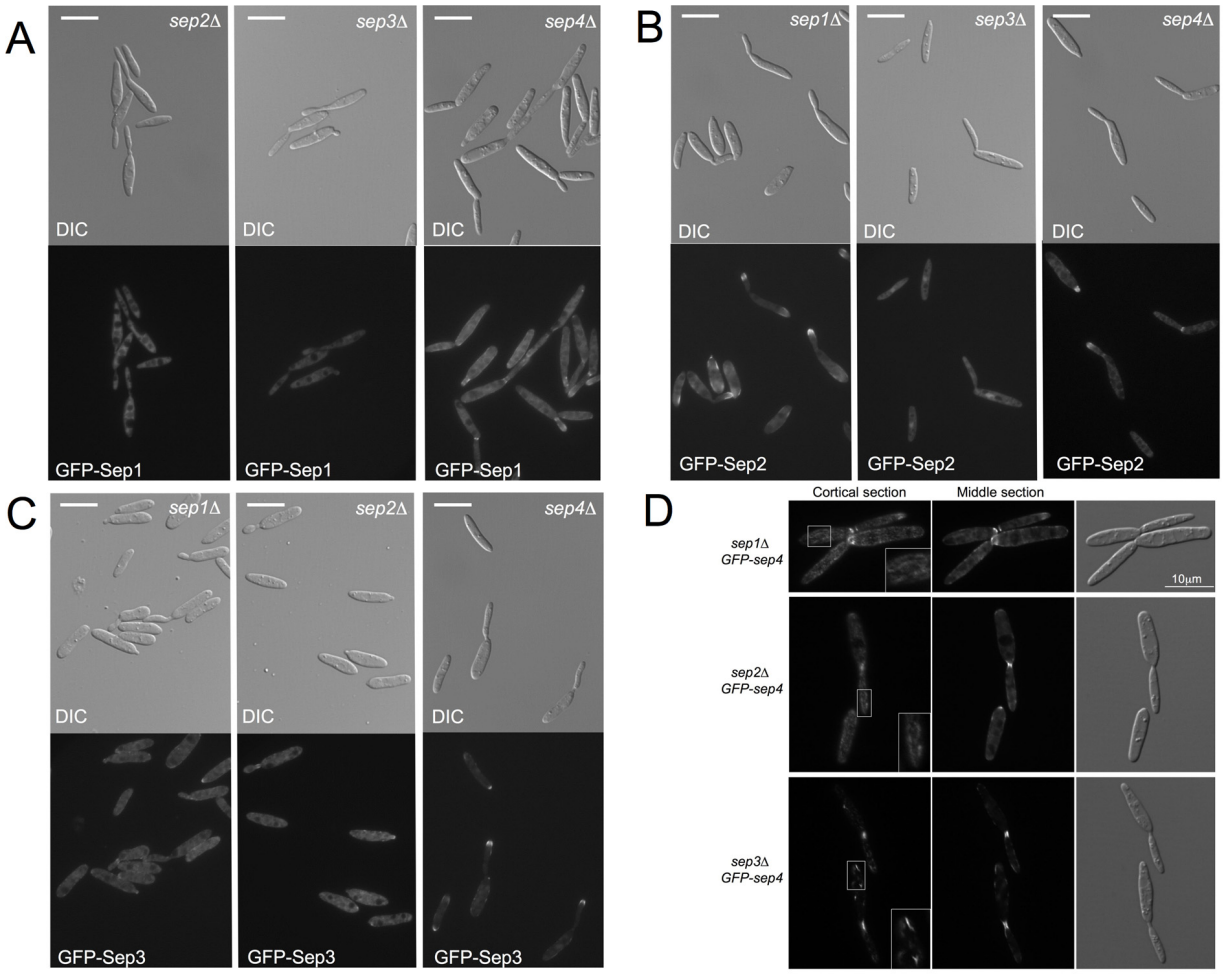
Septin dependencies in the formation of the different septin structures. A. GFP-Sep1 localization in *sep2*Δ, *sep3*Δ and *sep4*Δ mutant backgrounds. B. GFP-Sep2 localization in *sep1*Δ, *sep3*Δ and *sep4*Δ mutant backgrounds. C. GFP-Sep3 localization in *sep1*Δ, *sep2*Δ and *sep4*Δ mutant backgrounds. D. GFP-Sep4 localization in *sep1*Δ, *sep2*Δ and *sep3*Δ mutant backgrounds. Sep4 fibers were not observed in any of these strains suggesting that they required the presence of the other septins to assemble. A non-organized cortical matrix was observed in *sep1*Δ and *sep2*Δ cells (insets). In *sep3*Δ cells short filaments were scattered close to the cell cortex (inset). In all cases cells were grown to log phase at 22°C.

GFP-Sep1 was dependent on Sep2 and Sep3 for its localization at the bud neck as well as at the bud tip. In these mutant backgrounds GFP-Sep1 signal showed no specific localization other than cytoplasmic (Table 1). However, the absence of Sep4 did not affect bud neck nor bud tip localization (Fig. 6A, Table 1).

**Table 1:** Localization of Sep1-GFP fusion in different septin mutant backgrounds.

Localization	Number of cells (%)			
	wt	*sep2*Δ	*sep3*Δ	*sep4*Δ
Bud neck	72.2±1.2	0	0	63.2±0.8
Bud tip	36.5±1.7	0	0	50.4±1.2
Nuclear	0	0	0	0
mislocalized^1^	0	0	0	3.1±1.9
Non-specific^2^	27.8±1.2	100	100	33.7±2.4

A total of more than 100 cells per experiment (3 independent experiments) were counted per each mutant phenotype (p = 0.001).

1 Accumulation of signal outside of bud neck, bud tip or nuclear localization. For instance in cell walls.

2 Diffuse signal in cytoplasm.

GFP-Sep2 needed Sep1 to be located at the bud neck but its localization at the bud tip was unaffected in *sep1*Δ mutants (Table 2). In contrast, the absence of Sep3 strongly affected both sub-cellular localizations. It is worth mentioning that in this case, a clear nuclear accumulation of GFP signal was observed. Whether this nuclear accumulation is a non-specific side-effect of lack of Sep3 or it is uncovering some unexpected role of septins in the nucleus, it would deserve further investigation. As it happened with GFP-Sep1, the absence of Sep4 did not affect any of the localizations of GFP-Sep2 (Fig. 6B, Table 2).

**Table 2:** Localization of Sep2-GFP fusion in different septin mutant backgrounds.

Localization	Number of cells (%)			
	wt	*sep1*Δ	*sep3*Δ	*sep4*Δ
Bud neck	70.4±1.4	2.8±0.7	4.9±0.1	72.7±1.0
Bud tip	41.6±1.7	38.1±0.6	2.4±0.8	33.7±0.2
nuclear	0	0	93.3±1.7	0
mislocalized^1^	0	21.2±1.8	0.7±0.9	0
Non-specific^2^	29.6±1.9	40.7±2.2	1.1±1.2	27.3±2.0

A total of more than 100 cells per experiment (3 independent experiments) were counted per each mutant phenotype (p = 0.001).

1 Accumulation of signal outside of bud neck, bud tip or nuclear localization. For instance in cell walls.

2 Diffuse signal in cytoplasm.

GFP-Sep3 was localized both at the bud neck and the bud tip in the absence of Sep1 and Sep2, albeit in a lower proportion of cells (Table 3). In addition misplaced localizations were also frequent (lateral with regard to bud emergence). The absence of Sep4 did not preclude the localization of GFP-Sep3 at the bud tip but a very low proportion of cells with GFP-Sep3 located at the bud neck were observed (Fig. 6C, Table 3).

**Table 3:** Localization of Sep3-GFP fusion in different septin mutant backgrounds.

Localization	Number of cells (%)			
	wt	*sep1*Δ	*sep2*Δ	*sep4*Δ
Bud neck	65.8±0.8	18.1±1.8	15.5±1.5	2.7±1.6
Bud tip	32.8±1.0	6.5±1.3	7.1±0.9	74.8±1.6
nuclear	0	0	0	0
mislocalized^1^	0	34.2±2.3	40.3±4.0	0
Non-specific^2^	34.2±0.8	47.7±2.6	44.2±4.9	25.2±1.5

A total of more than 100 cells per experiment (3 independent experiments) were counted per each mutant phenotype(p = 0.001).

1 Accumulation of signal outside of bud neck, bud tip or nuclear localization. For instance in cell walls.

2 Diffuse signal in cytoplasm.

Finally, we found that Sep4 localization at the bud neck and bud tip was unaffected by the absence of any of the other 3 septins (Fig. 6D). However, in the absence of any of the other septins, Sep4 fibers were not observed. It is worth mentioning that different outcomes were obtained depending on which septin was removed. Removal of Sep1 or Sep2 produced a cortical unorganized matrix, while removal of Sep3 resulted in the appearance of short filaments scattered throughout the cell, close to the cell cortex (Fig. 6D). We believe that the absence of Sep4 fibers in cells defective in Sep1–3 septins reflects a true requirement of these septins in the formation of the long Sep4 filaments: we were able to observe this impairment in filament formation at 22°C, conditions in which the mutant cell morphology seemed to be less affected, discarding that the inability to form long Sep4 filaments was an epi-phenomenon of the aberrant morphology observed in septin mutants at 28°C.

### Sep4 fibers were independent of the actin- and microtubule-cytoskeletons

**Figure 7:**
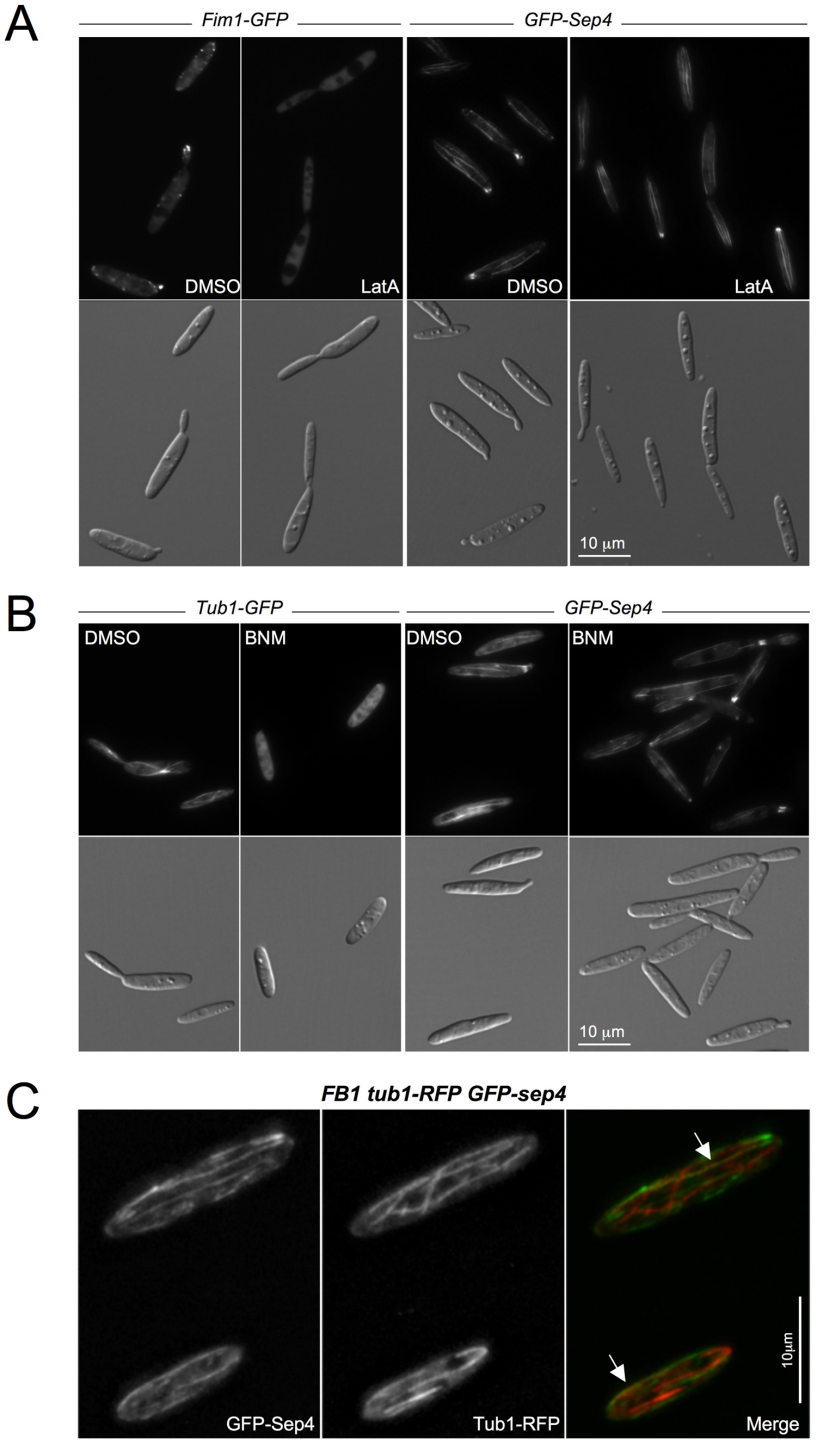
Sep4 filaments and the F-actin and microtubule cytoskeletons. A. Fim1-GFP and GFP-Sep4 cells were grown to log phase and treated for 10 minutes with Latrunculin A (LatA, 10 µM) or its solvent DMSO. Fim1-GFP localization to F-actin patches was lost upon LatA treatment but GFP-Sep4 filaments were present as they were upon solvent treatment. B. Tub1-GFP and GSP-Sep4 cells were grown to log phase and treated for 20 minutes with Benomyl (BNM; 30 µM) or its solvent DMSO. Microtubule depolymerisation upon BNM treatment did not affect the maintenance of GFP-Sep4 filaments. C. Tub1-RFP GFP-sep4 cells were grown to log phase and red and green filter sets were used to capture sequential images over the complete volume of the cells. DeltaVision deconvolved images are shown alongside a Merge image. A partial co-alignment of Sep4 and microtubules was observed (arrow).

In mammalian cells, higher-order septin organization has been linked to both the F-actin [36], [37] and the microtubule cytoskeletons [38]. We were curious about whether Sep4 fibers showed any relationship with either the F-actin- or microtubule-based cytoskeletons in *U. maydis*. Firstly, we perturbed the normal organization of the F-actin or microtubule cytoskeletons by drug treatments and analyzed their effects on the Sep4 fibers. We incubated *GFP-sep4* cells with the F-actin-depolymerising drug Latrunculin A (LatA) [12], [39], [40], the microtubule-destabilizing drug benomyl (BNM) [35], [40], or their solvent dimethyl sulfoxide (DMSO). In conditions that produced the disorganization of the F-actin or microtubule cytoskeletons (Figs. 7A and 7B) -we used as control a strain expressing either Fim1-GFP [39] or GFP-Tub1 fusions [41]-, no differences were observed between the Sep4 fibers of drug-treated cells and solvent-treated cells, indicating that the maintenance of the Sep4 fibers was independent of the F-actin and microtubule cytoskeletons. We also analyzed the F-actin and microtubule cytoskeletons in the absence of *sep4*. No differences were appreciable in these cytoskeletons between wild-type and *sep4*Δ cells (Fig. S2). Although we cannot exclude the possibility of subtle differences in the assembly or dynamics of actin and microtubules between wild-type and mutant strains, it seems that the actin- and microtubule-cytoskeletons were unrelated to Sep4 fibers.

Even when microtubules and septin fibers seemed to be independent of each other, since they run along the long cell axis we were curious about the distribution of septin fibers in relation to the microtubule cytoskeleton. To analyze this we introduced in GFP-Sep4 cells a construction expressing a RFP-Tub1 fusion [42]. We found in a high percentage of the cells (78% of cells, n = 45) a subset of septin fibers co-localizing with microtubules (Fig. 7C). Co-localization of septins with microtubules in fungi has been previously noted in *S. cerevisiae* cells growing during nutrient limitation [43] as well as in *C. neoformans* [16]. Whether this co-localization reflects functional connections between septins and microtubules is currently unclear and awaits further experimentation.

### Septins are dispensable for virulence

**Figure 8:**
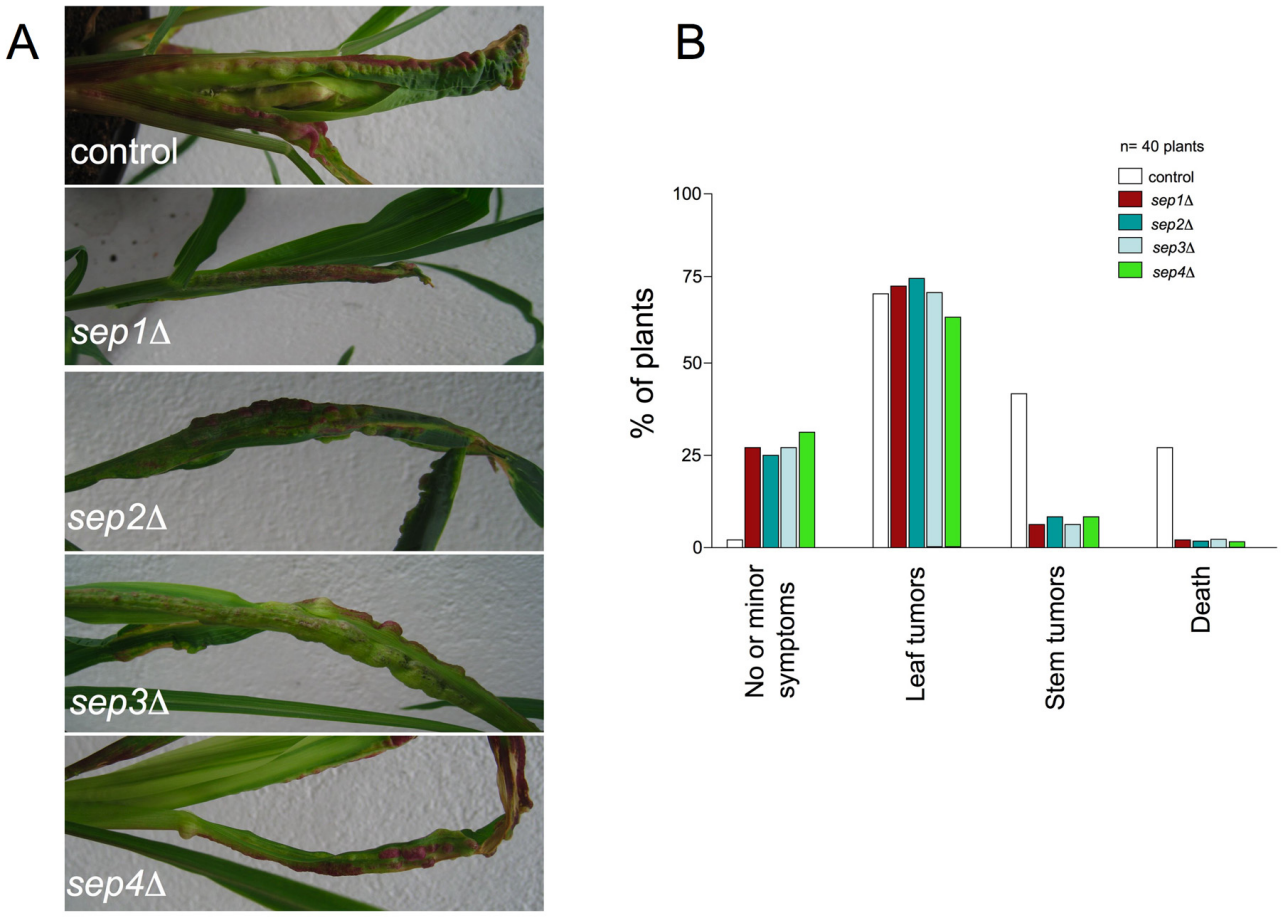
Virulence of septin mutants. A. Leaves of plants inoculated either with wild-type or septin mutant strains. B. Quantification of tumor formation on infected maize plants

*U. maydis* infection of maize results in the formation of tumors that are filled with proliferating fungal cells that eventually differentiate into black teliospores [44]. To determine the importance of septins in virulence, we infected maize plants with a mixture of compatible control strains FB1 and FB2 as well as the mutant strains. A previous report showed that *sep3* mutants were less virulent than wild-type strains when they were injected at low inoculum concentration [15]. Therefore, we used a low inoculum concentration (10^5^ cells) in our assays. We observed that all septin mutants were able to infect corn plants, although they showed attenuated symptoms. For instance, few mutant infections produced stem tumors, while leaf tumors were observed at a similar ratio as in wild-type control infections (Fig. 8A, B). Mutant strains were also able to produce teliospores (see below).

**Figure 9:**
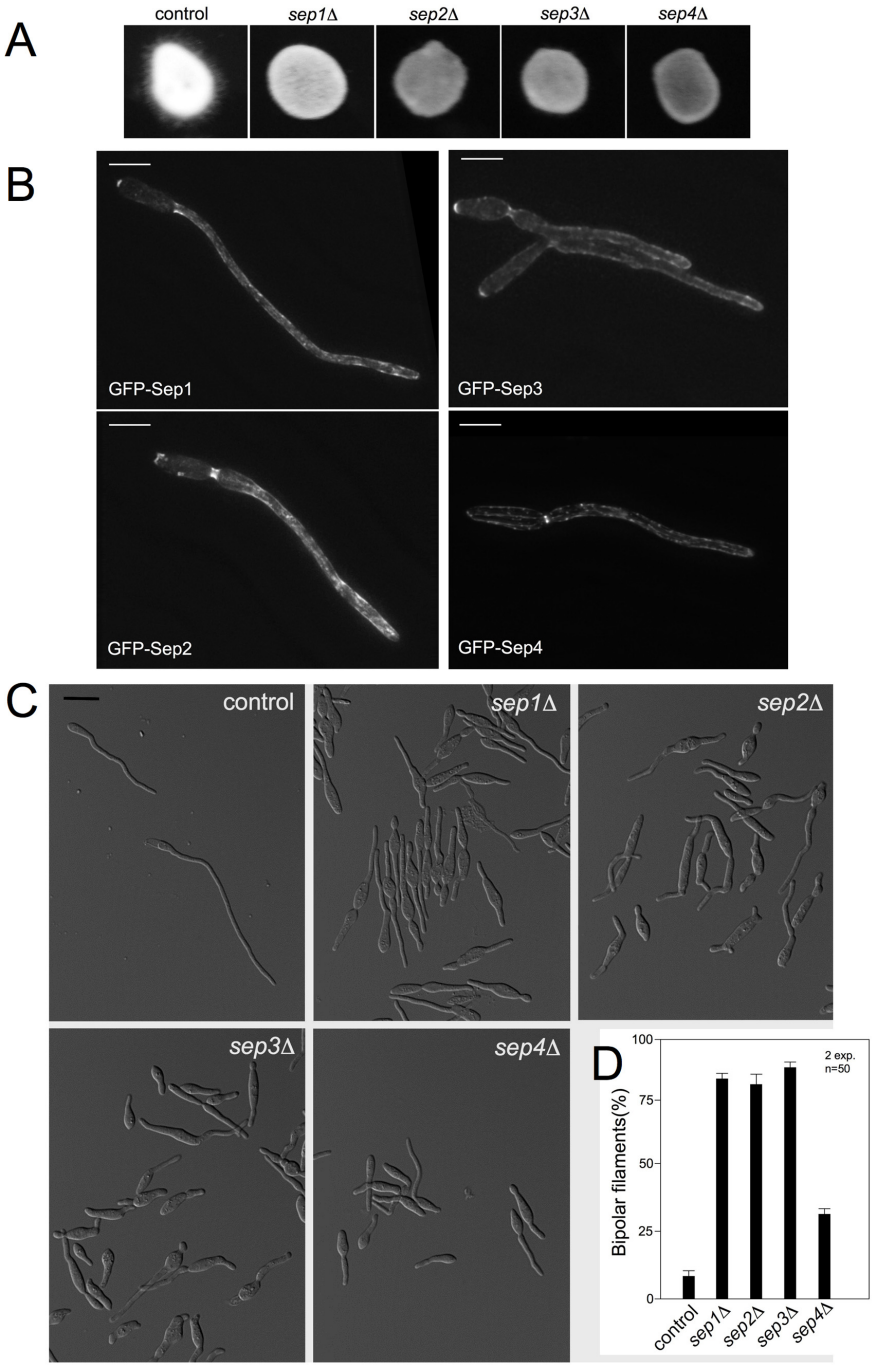
Septins are required for the proper formation of *b*-dependent filaments. A. Crosses of control strains FB1 X FB2 and septin mutant strains in charcoal-containing agar plates. Note the gray appearance of mutant crosses indicating impairment in filament formation. B. Septin localization in *b*-dependent filaments. GFP-septin alleles were introduced into AB31 strain, which carries compatible *bE* and *bW* alleles under the control of *crg1* promoter and forms *b-*dependent filaments upon shift to minimal medium with arabinose as carbon source. Images were obtained after 6 h of incubation in inducing conditions. C. Morphology phenotype of septin null mutants in AB31 background. Observe that septin null mutants showed impaired hyphal growth after 6 hours of incubation in inducing conditions, as well as bipolar filamentation. Bar: 15 µm. D. Graph indicating the percentage of cells that grew bipolarly 6 hours after filament induction. Error bars indicate s.d., more than 50 filaments were analysed for each strain.

In order to infect corn plants, *U. maydis* relies on a dramatic morphogenetic change. Plant infection by *U. maydis* requires the formation of an infective filament, which invades the epidermis and continues the pathogenic program inside the plant. The production of this infective filament is linked to a mating process that, after cell fusion, leads to the interaction of the two subunits composing the transcription activator b-factor, bW and bE, each one provided by each mating partner [45]. The formation of dikaryotic filaments can be easily monitored on charcoal-containing agar plates [46]. As we were surprised by the absence of a much higher effect in virulence when septins were absent, we wondered whether the infective filament was affected. For this, we crossed compatible wild-type strains and septin defective strains onto charcoal-containing plates. Wild-type crosses led to a typical fuzzy colony appearance, which was attributable to the massive formation of dikaryotic hyphae [47]. In contrast, septin mutants were attenuated in filament formation (Fig. 9A).

To investigate this filament formation defect in more detail, we made use of the previously described haploid *U. maydis* AB31 strain, which carries compatible *bE* and *bW* genes under the control of the regulatable *crg1* promoter [48]. In this strain, *b*-dependent infective filament formation can be elicited by incubating the cells in a medium containing arabinose as carbon source. In the first place, we analyzed septin structures during the infective filament formation using the GFP-tagged alleles introduced into AB31. Our results showed septin localization at the neck as well as at the filament tip. In addition, septin filaments were observed scattered throughout the filament. Moreover, a clear accumulation of fluorescence of GFP-Sep1-3 fusions was observed at the distal pole of filament emergence (Fig. 9B). It is noteworthy that at the distal pole septa leaving empty spaces are formed as growth of the filament proceeds [49], so it is possible that the observed basal septin accumulation would be related to the formation of these septa.

We also introduced the different septin deletion alleles in AB31 cells and analyzed their cellular involvement in *b*-dependent polar growth. We observed that septin mutant cells were disturbed in *b*-dependent polar growth (Fig. 9C). Filament formation was retarded, although after 12 hours of induction mutant cells reached the typical maximal length of the living tip cell observed in the control strain (around 90 µm, not shown). In addition, all septin mutant cells produced bipolar growing filaments. The percentage was around 80% of the cells in *sep1-3* mutant strains and around 30% in *sep4* mutant strain (Fig. 9D). Whether this result is related to the above described presence of septins at basal locations is not known, although is an appealing possibility.

### Germination of teliospores is affected in septin mutant strains

**Figure 10:**
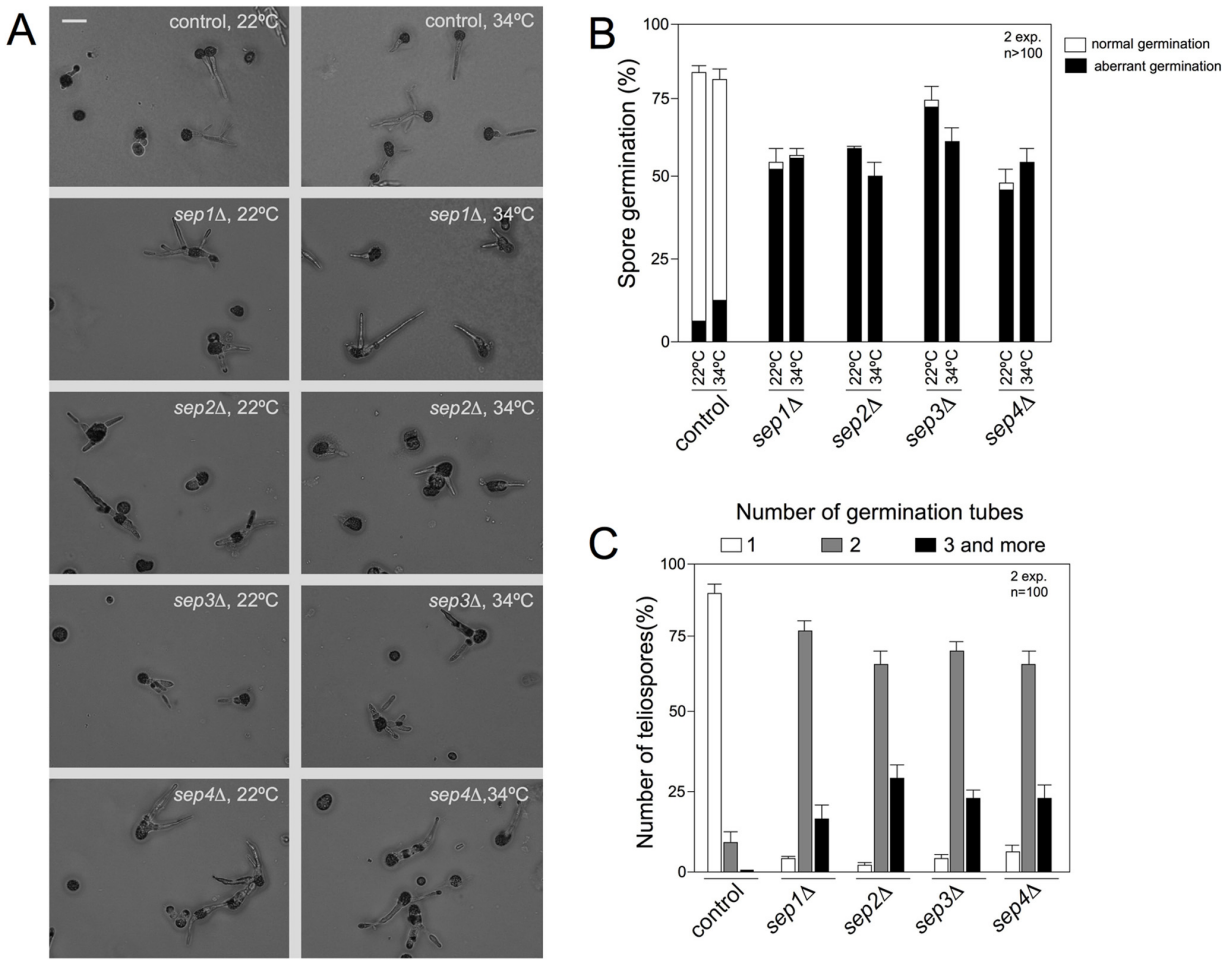
Septins are required for the proper germination of teliospores. A. Images of control and septin mutant teliospores germinated on CM-glucose-containing agar slides after 24 h of incubation at 22°C and 34°C. Note that wild-type teliospores extend a promycelium, from which haploid progeny are generated. In mutant teliospores aberrant germination is observed. Bar: 20 µm. B. Graph indicating the percentage of teliospores able to germinate after 24 h at two different temperatures (22°C and 34°C). Teliospores which germination produced germ tubes swelled or that were abnormal in shape, as well as teliospores producing more than one promycelium were considered as aberrant. Error bars indicate s.d., more than 100 teliospores were analysed for each strain. C. Graph indicating the percentage of teliospores displaying one or more than one germination tube after 24 h at 22°C. Error bars indicate s.d., 100 teliospores were analysed for each strain.

As we mentioned above, septins were dispensable for virulence in *U. maydis* and plants infected by septin mutants developed tumors that eventually were filled by melanized diploid teliospores. In the field, germination of the air-borne diploid teliospores is the first step in the infection process and therefore germination of teliospores is required to fulfill the life cycle in this fungus. *U. maydis* teliospore germination is a complex process that includes a switch from dormancy to physiological activity, the rupture of the thick cell wall, extension of a tubular promycelium and the completion of meiosis to produce haploid cells. Emergence of the promycelium implies the establishment of a new polarity axis, and therefore a role of septins in this process could be predicted. In fact, a previous report already described defects in germination of teliospores obtained from *sep3* mutant strains [15]. To extend these observations to the other septins, collected tumors from infected plants with wild-type or septin mutant cells were ground and teliospores isolated. Teliospores preparations were plated onto complete medium agar-coated slides and incubated for 24 hours at two different temperatures (22°C and 34°C) to observe and quantify teliospore germination (Figs. 10A and 10B). Wild-type teliospores germinated by extending a promycelium, with subsequent meiosis and the formation of haploid progeny as buds from the promycelium (Figure 10A, control). However, although a substantial proportion of septin mutant teliospores were able to germinate at both temperatures (ranging between 50 and 75%, Fig. 10B), they showed abnormal morphology including swelling of promycelium and aberrant shape. Also it was noticeable that all septin mutants produced more than one germination tube per germinated teliospore at both temperatures (Fig. 10C and not shown). The proportion of this defect was 90% in average for the mutants at both temperatures and 6% (22°C) and 11% (34°C) in wild-type teliospores (Fig. 10C and not shown).

In spite of these defects during germination, mutant teliospores were able to produce haploid progeny (not shown).

## Discussion

This paper investigates the role of septins in the life cycle of *U. maydis* and comes to three main conclusions. The first one concerns to the presence of three distinct septin structures coexisting in the same cell in *U. maydis*, which were observed using functional GFP-tagged alleles. Two of these structures, located at the bud neck and the bud tip, were similar to other structures already described in fungi, while the third one, fibers running from pole to pole, has been less described. The second finding relates to the role that septins may have in morphogenesis in *U. maydis*. We observed that although not essential for growth, mutant cells lacking septins display an aberrant morphology that cannot be explained simply invoking a defect in bud neck formation, arguing additional roles of septins during morphogenesis in *U. maydis*. Finally, our third main conclusion refers to the ability of septin mutants to infect plants that contrasts with the impaired virulence of septin mutants described in other pathogenic fungi. Our discussion briefly reviews our evidence for these conclusions and places them in context with other works addressing the role of septins in fungal morphogenesis and virulence.

### *U. maydis* cells assemble three different septin structures

Many studies addressed the subcellular localization of septins in a wide range of fungi and animals. A recent review [6] made the interesting proposal that subcellular septin localization falls into variations of three distinct patterns, which correlates with the proposed function: localization to partitions, to projections or to whole cells. The structures we found in *U. maydis* fall into the two last patterns: two of them are located at projections (bud) - either the base of the bud or at the tip of the bud- and the third one is located as a septin fiber throughout the whole cell. Since *U. maydis* divides by budding, the finding that septins localized at the bud neck was somehow expected. We found that the four septins localized as a tight ring that appeared just before bud emergence. The bud then grew out through the septin ring, which later on appeared as doublet and finally decreased in intensity concurrent with mother daughter cell separation. A recent report [50] showed that in *U. maydis*, this septin structure at the bud neck was controlled by the kinase Don3, a master regulator of cytokinesis in *U. maydis*. Therefore, we assumed that the main role of septins located at the bud neck has to be related to cytokinesis, playing a similar role as it does in *S. cerevisiae*.

Less expected was the localization of septins as a band-like structure behind the bud tip. Although this localization has been described in some mutant backgrounds in *S. cerevisiae* [23]–[29], in wild-type conditions it has been described at the tip of filamentous fungi such as *Aspergillus nidulans* and *Ashbya gossypii* [4], [51] as well as at the tip of the germ tube in *C. albicans* during dimorphic switch [22], [52], [53]. It is worth mentioning that bud growth in *U. maydis* is supported by a continuous polar growth during G2 phase in contrast to the isotropic growth observed during G2 phase in budding yeast. In this sense, bud growth in *U. maydis* resembles filamentous fungi tip growth. We believe that the apical localization of septins in *U. maydis* reflects a role of septins in the polar growth machinery of *U. maydis*. It is thought that during filamentous growth, septins may play a role by facilitating the organization of specific plasma membrane domains, such as sterol-rich lipid rafts [8]. Interestingly, previous work done in our laboratory showed that in *U. maydis* this band-like septin structure partially overlaps with a filipin-stained ergosterol-rich region of the plasma membrane at bud tips [54].

The third septin structure we found consisted of fibers running from pole to pole of the cell near the cortex. We are confident that these filaments represent a physiological structure. An argument to support these fibers is that we found the same structures in number and appearance when using immunofluorescence of a pk-tagged version of Sep4. The ability of septins to form long fibers was firstly described in *C. albicans* chlamydospores [55] and it has been recently described in other fungi. For instance, in *A. gossypii* the septin Sep7 seems able to form very thin and long cortical filaments that run parallel to the growth axes of the hyphae [56]. Also, in *C. neoformans,* Cdc10-mcherry fusions formed filaments along the hyphal cell [16]. It is unclear the role that these structures may have in fungal cells. Sep4 fibers seem to be independent of the F-actin- and microtubule- cytoskeletons. However, we cannot discard the existence of some relationships between septins and these cytoskeletons, particularly with microtubules as it is possible to observe cells with a subset of septin and microtubule filaments co-aligned.

A striking result we obtained refers to the ability of some of the septins to remain as part of higher-order structures in the absence of other septins. In *S. cerevisiae*, for instance, the septin ring is typically disrupted when one septin gene is deleted [19], [20], [21], [57] and similar findings were reported in *C. neoformans* [16] and *A. gossipy* [58]. However, this is not always the case, and our results agree with reports in *C. albicans* [22] and *Schizosaccharomyces pombe* [59] about persistence of septin structures in the absence of some subunits. In our case, the absence of Sep4 seems to have the less deleterious effect on other septins localization, affecting only to the bud neck localization of Sep3. In contrast, the lack of the other septins affected more broadly the localization of the rest of septins, with the exception of Sep4 that was affected only in its ability to form fibers. From our results it looks like there are two groups with respect to this interdependence: one group is composed of septins Sep1-3, while the second one is composed of Sep4. Interestingly, no pairwise combination between sep1-3 was lethal while two of the combinations including sep4 were lethal. The general model for the formation of septin polymers in yeast suggests that Cdc10 links polymeric septin rods together [60]. Accordingly, it is interesting that Sep4, the Cdc10 homologue, appears to have the least important role in affecting the localization of other *U. maydis* septins. Whether these relationships reflect the ability of septins to produce distinct subcomplexes is currently unknown, and it will require additional research.

### Septins are required for proper morphogenesis in *U. maydis*

None of the *U. maydis* septins seem to be essential although the disruption of any septin gene produces morphological defects that are exacerbated with the temperature and show a terminal phenotype at 34°C with more than 90% of cells losing all polarity and lysing. This enhancement can be also obtained when cells were grown at low temperature and treated with BFA, which impairs exocytosis. These results together with the higher sensitivity of septin mutants to drugs affecting cell wall strongly support the idea that in *U. maydis* septins are required for an efficient cell wall construction. Strikingly, the presence of sorbitol not only avoided the cell lysis but also recovered the morphology of the mutant cells. One appealing possibility could be that higher order septin structures in *U. maydis* serve as a guide of material distribution, enabling the even delivery of cell wall material. Septins have been proposed to be involved in exocytosis in mammalian cells. For example, septins have been found to associate with the sec6/sec8 exocyst complex in rats [61] and also with synaptic vesicles in mice [62], suggesting that they might participate directly in vesicle trafficking and regulated secretion. In this scenario, one of the consequences of septin deletion in *U. maydis* would be a less solid cell wall unable to support physical pressure as a consequence of an impaired delivery of cell wall material.

### Septins play a minor role during virulence

In agreement with a previous report about Sep3 [15], we found that none of septin mutants were severely affected in virulence. This result contrasts with the recent characterization of septins in *C. neoformans*, which is also a pathogenic basidiomycete fungus, describing a more important effect of septin deletion in virulence assays [16]. Also in *C. albicans*, virulence was impaired in septin mutants [17]. In spite of the absence of a more dramatic effect in virulence by ablation of septin function, we concentrated our efforts to determine whether absence of septins would affect the formation of the infective filament in *U. maydis.* We observed two major defects during the formation of the infective filaments in strains defective in septins: the filament elongation was retarded in comparison to wild-type strains, and a higher proportion of the cells grew in a bipolar manner. None of these defects seem to disable fungal cells to infect plants. Mutations that affected the ability to elongate the infective filament at normal speed or that produce bipolar filamentation have been previously described in *U. maydis*. Cells defective in Pcl12, a Cdk5-specific cyclin are impaired in polar growth during filament formation, but are still able to infect plants [14]. In the same way, cells defective in the RNA-binding protein Rrm4 also show defects in elongation and a high level of bipolar growth but they are still able to form tumors [63], [64]. It is worth mentioning that in all these cases, even when infection was successful and tumors were produced, they were rarely present in stem while leaf tumors were often observed. A naive explanation is that decreasing the efficiency to produce an elongated infective filament could affect the ability to navigate through the plant surface to locate at more distal points of vulnerable sites for infection, and therefore the infection takes place in sites near to the inoculation point, which used to be basal leaves.

Finally, we confirmed and extended to the other septins the previous observation that teliospore germination was affected by the absence of Sep3 [15]. Although mutant teliospores were able to germinate and to produce haploid progeny, they showed aberrant germination, with frequent swelling of germinative tube as well as the emergence of more than one germinative hypha. A recent report about septin function during germination of conidiospores in *A. nidulans*, indicated that septins are important for preventing the inappropriate emergence of germ tubes and branches [65]. To do that, *A. nidulans* septins localized as discrete spots in dormant and expanding conidia, as well as rings at forming septa and at the bases of emerging germ tubes. Although preliminar, we observed GFP-septin fusion in germinating teliospores (Fig. S3) and found that these fusions were visible as spots in dormant conidia as well as at the tip of germ tube and forming part of septa. Whether this localization reflects the roles adscribed to septins in *A. nidulans* conidiospores germination will require additional experimentation.

## Materials and Methods

### Strains and growth conditions

*Ustilago maydis* strains are derived from FB1 and FB2 genetic backgrounds [47] and are listed in Table S1. Cells were grown in rich medium (YPD), complete medium (CM) or minimal medium (MM) [46]. Inhibitor studies were performed as described previously [12], [35], [40].

### Plasmid and strain constructions

Plasmid pGEM-T easy (Promega) was used for cloning, subcloning and sequencing of genomic fragments and fragments generated by PCR. Sequence analysis of fragments generated by PCR was performed with an automated sequencer (ABI 373A) and standard bioinformatic tools. To construct the different strains, transformation of *U. maydis* protoplasts with the indicated constructions was performed as described previously [66]. *U. maydis* DNA isolation was performed as previously described [66]. The following fusions were already described: GFP-Tub1 [41], RFP-Tub1 [67], Fim1-GFP [39], and NLS-GFP [68].

Deletion of each septin gene was done by gene replacement following published protocols [69]. Briefly, a pair of DNA fragments flanking the corresponding septin ORF were amplified and ligated to hygromicin, nourseotricine or caboxin resistance cassettes via *Sfi*I sites. The 5′ and 3′ fragments were amplified using the oligonucleotide pairs SEPn-1/SEPn-2 and SEPn-3/SEPn-4 (where n varies from 1 to 4 depending on the septin, Table S2) respectively. Each fragment was about 1 kbp in length. Integration of the disruption cassette into the corresponding loci was verified in each case by diagnostic PCR and subsequent Southern blot analysis.

For subcellular localization N-terminal GFP-septin fusions were produced. For this, an *Nde*I site was introduced at the ATG of each septin locus using PCR-based two-step mutagenesis protocol (amplification was performed using the primer pairs: SEPn-10/SEPn-11 and SEPn-12/SEPn-13). A stopless *Nde*I GFP cassette was introduced at the respective *Nde*I site and the GFP-septin fusion integrated by homologous recombination under their respective native septin promoter.

### Plant infections, mating assays and germination of teliospores

Pathogenic development of wild type and mutant strains was assayed by plant infections of the maize (*Zea mays*) variety Early Golden Bantam (Olds seeds) as described [15]. For charcoal mating assays, strains were crossed on charcoal-containing complete medium plates and incubated at 22°C [46].

To assay the germination of teliospores, infected plants were incubated for 20 days. Tumors containing spores were dried at room temperature and minced using a mortar and pestle. The tumor material was incubated overnight in a 1.5% copper (II) sulfate. After extensive washing in sterile distilled water, spores were plated on 2% complete medium-containing agar slides and incubated in a moist chamber at 22°C or 34°C [14].

### Microscopy

Images were obtained either using a Nikon Eclipse 90i fluorescence microscope with a Hamamatsu Orca-ER camera driven by Metamorph (Universal Imaging, Downingtown, PA) or a DeltaVision RT Restoration Microscopy System with a Coolsnap HQ camera driven by SoftWoRx v.3.5.0 Software. One focal plane images are shown unless otherwise specified. Nikon Eclipse images are shown unless otherwise specified. Images were further processed with Adobe Photoshop CS or Imaris 6.0.1 software.

## Supporting Information

Figure S1 Immunolocalization of Sep4. We constructed a strain expressing an N-terminal pk-tagged version of Sep4 under its own promoter. For indirect immunofluorescence we adapted a procedure kindly provided by Prof. G. Steinberg. Briefly, formaldehyde (EM-grade, Polyscience) was added to growing cultures to a final volume of 4% and cells were fixed for 30 minutes, washed with phosphate- buffered saline (PBS, pH 7.2) and applied to coverslips pre-coated with poly-L-lysine (Sigma). This was followed by washes with PBS and 30 minutes of treatment with 3 mg/ml Novozyme (NovoNordisk). Subsequently cells were washed and incubated in 1% Triton X-100 for 30 sec, followed by additional washes and incubation in blocking reagent (2% milk powder, 2% BSA in PBS, pH 7.2) for 10 minutes. Antibodies against the pk epitope (a gift of Prof. Iain Hagan, Manchester, UK; Craven et al., 1998) were diluted (1∶25) in 0.2% milk, 0.2% BSA, 0.01% azide in PBS, pH 7.2 and applied overnight at 4°C. After several washes, samples were incubated with diluted (1∶500) secondary antibody (goat anti-mouse Alexa-fluor 488, Invitrogen A11029) for 2–3 h at room temperature. After 5 final washes with PBS, pH 7.2, samples were mounted and observed under the microscope. The image (A) shown is composed from images taken from different fields and assembled using Photoshop. Bar: 15 µm. In (B) quantification of number of fibers per cell is shown. Serial Z-images were obtained per each cell and maximal projections were used to determine the number of fibers per cell. Craven RA, Griffiths DJ, Sheldrick KS, Randall RE, Hagan IM, Carr AM. (1998) Vectors for the expression of tagged proteins in Schizosaccharomyces pombe. Gene 221: 59-68.
(0.69 MB TIF)

Figure S2 Actin and microtubule cytoskeletons in sep4Δ cells. (A) sep4Δ tub1-GFP and tub1-GFP cells were grown to log phase at 22°C. Micrographs showed that the microtubule cytoskeleton of sep4Δ cells was similar to the wild-type one. (B) sep4Δ fim1-GFP and fim1-GFP cells were grown to log phase at 22°C. Micrographs showed that the F-actin cytoskeleton was not affected by the absence of Sep4.
(0.45 MB TIF)

Figure S3 Septin localization in germinating teliospores. Corn plants were infected with crosses of compatible strains carrying the GFP-tagged septin alleles indicated. Teliospores were obtained and germinated on 2% complete medium-containing agar slides and incubated in a moist chamber at 22°C. Figures are composed images from DIC as well as epifluorescence individual images. A non-germinated teliospore can be observed as well as two distinct stages during germination process. Bar: 20 µm.
(2.06 MB TIF)

Table S1 U. maydis strains used in this study.
(0.09 MB DOC)

Table S2 Oligonucleotide primers used in this study.
(0.06 MB DOC)

Movie S1 Sep4 fibers. GFP-Sep4 cells were grown to log phase at 22°C and 15 z-series of 0,2 µm focal-plane steps were acquired in a DeltaVision System. Imaris 6.0.1 software was applied to generate a movie.
(8.20 MB MOV)
