# Supplementary figures and images for: Septins from the Phytopathogenic Fungus Ustilago maydis Are Required for Proper Morphogenesis but Dispensable for Virulence
Source: PLoS One. 2010 Sep 27;5(9):e12933. doi: 10.1371/journal.pone.0012933 (PMC2946335; doi:10.1371/journal.pone.0012933)

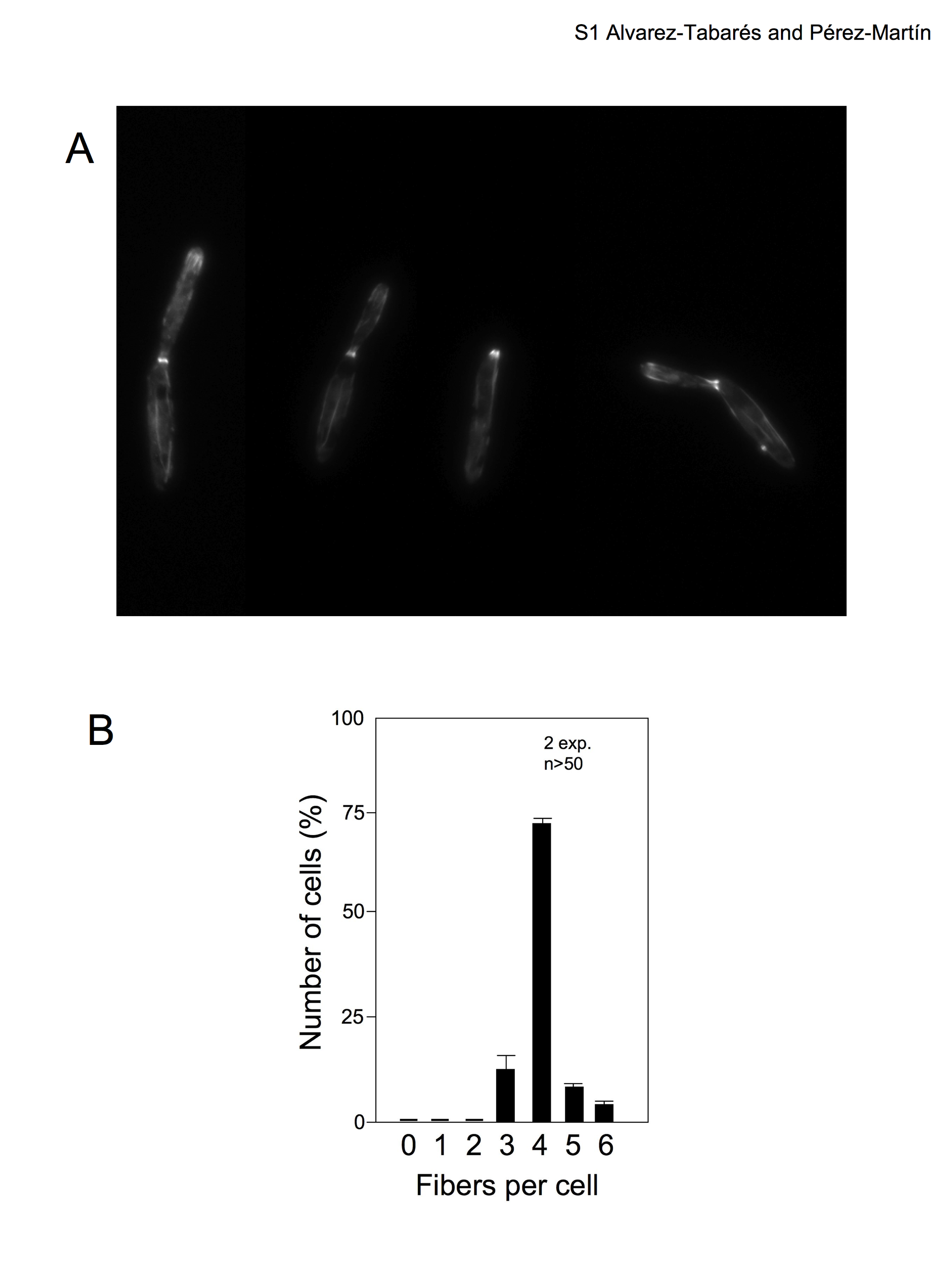

Supplement: Figure S1 — Immunolocalization of Sep4. We constructed a strain expressing an N-terminal pk-tagged version of Sep4 under its own promoter. For indirect immunofluorescence we adapted a procedure kindly provided by Prof. G. Steinberg. Briefly, formaldehyde (EM-grade, Polyscience) was added to growing cultures to a final volume of 4% and cells were fixed for 30 minutes, washed with phosphate- buffered saline (PBS, pH 7.2) and applied to coverslips pre-coated with poly-L-lysine (Sigma). This was followed by washes with PBS and 30 minutes of treatment with 3 mg/ml Novozyme (NovoNordisk). Subsequently cells were washed and incubated in 1% Triton X-100 for 30 sec, followed by additional washes and incubation in blocking reagent (2% milk powder, 2% BSA in PBS, pH 7.2) for 10 minutes. Antibodies against the pk epitope (a gift of Prof. Iain Hagan, Manchester, UK; Craven et al., 1998) were diluted (1∶25) in 0.2% milk, 0.2% BSA, 0.01% azide in PBS, pH 7.2 and applied overnight at 4°C. After several washes, samples were incubated with diluted (1∶500) secondary antibody (goat anti-mouse Alexa-fluor 488, Invitrogen A11029) for 2–3 h at room temperature. After 5 final washes with PBS, pH 7.2, samples were mounted and observed under the microscope. The image (A) shown is composed from images taken from different fields and assembled using Photoshop. Bar: 15 µm. In (B) quantification of number of fibers per cell is shown. Serial Z-images were obtained per each cell and maximal projections were used to determine the number of fibers per cell. Craven RA, Griffiths DJ, Sheldrick KS, Randall RE, Hagan IM, Carr AM. (1998) Vectors for the expression of tagged proteins in Schizosaccharomyces pombe. Gene 221: 59-68. (0.69 MB TIF) [file pone.0012933.s001.tif]

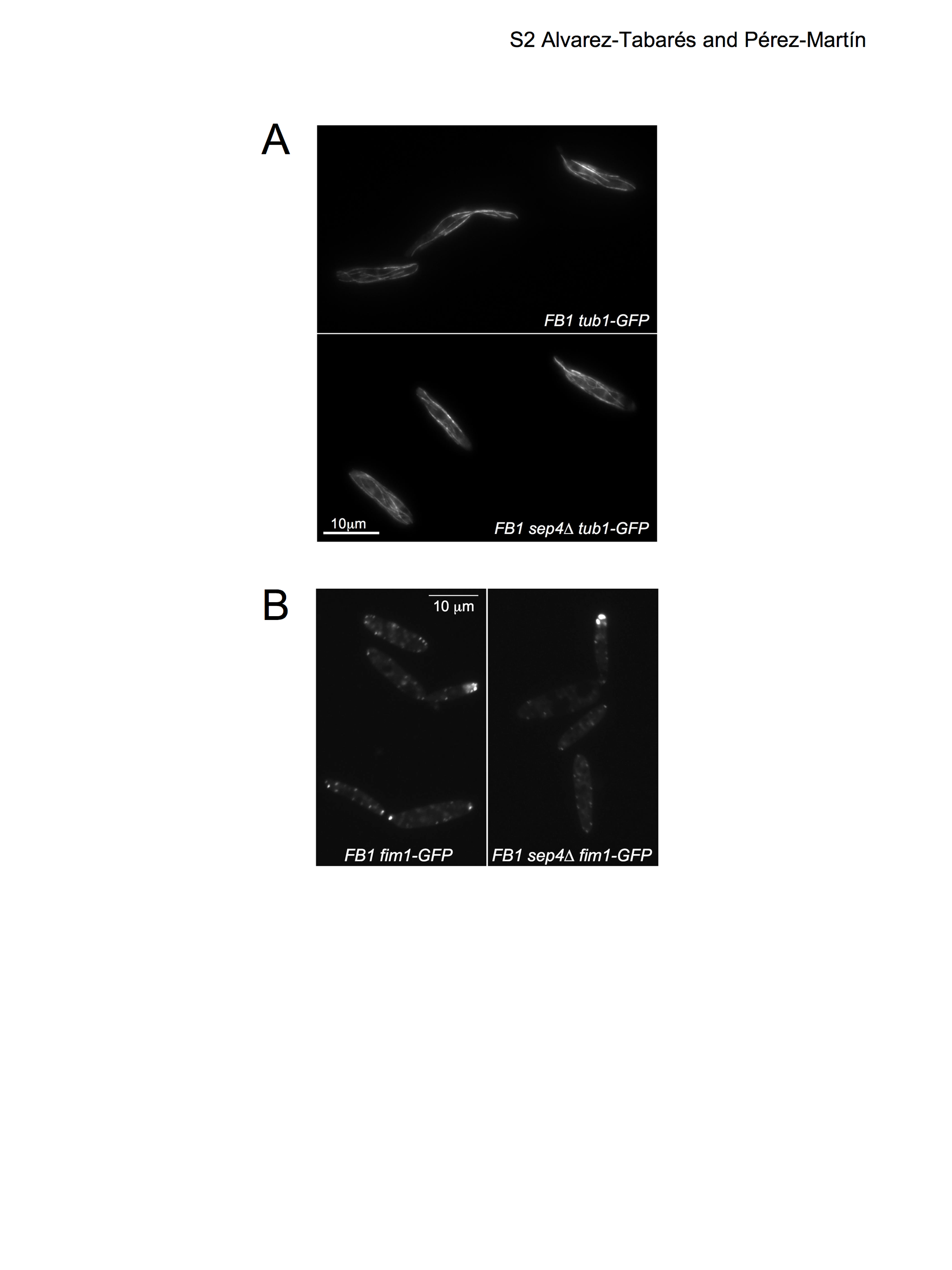

Supplement: Figure S2 — Actin and microtubule cytoskeletons in sep4Δ cells. (A) sep4Δ tub1-GFP and tub1-GFP cells were grown to log phase at 22°C. Micrographs showed that the microtubule cytoskeleton of sep4Δ cells was similar to the wild-type one. (B) sep4Δ fim1-GFP and fim1-GFP cells were grown to log phase at 22°C. Micrographs showed that the F-actin cytoskeleton was not affected by the absence of Sep4. (0.45 MB TIF) [file pone.0012933.s002.tif]

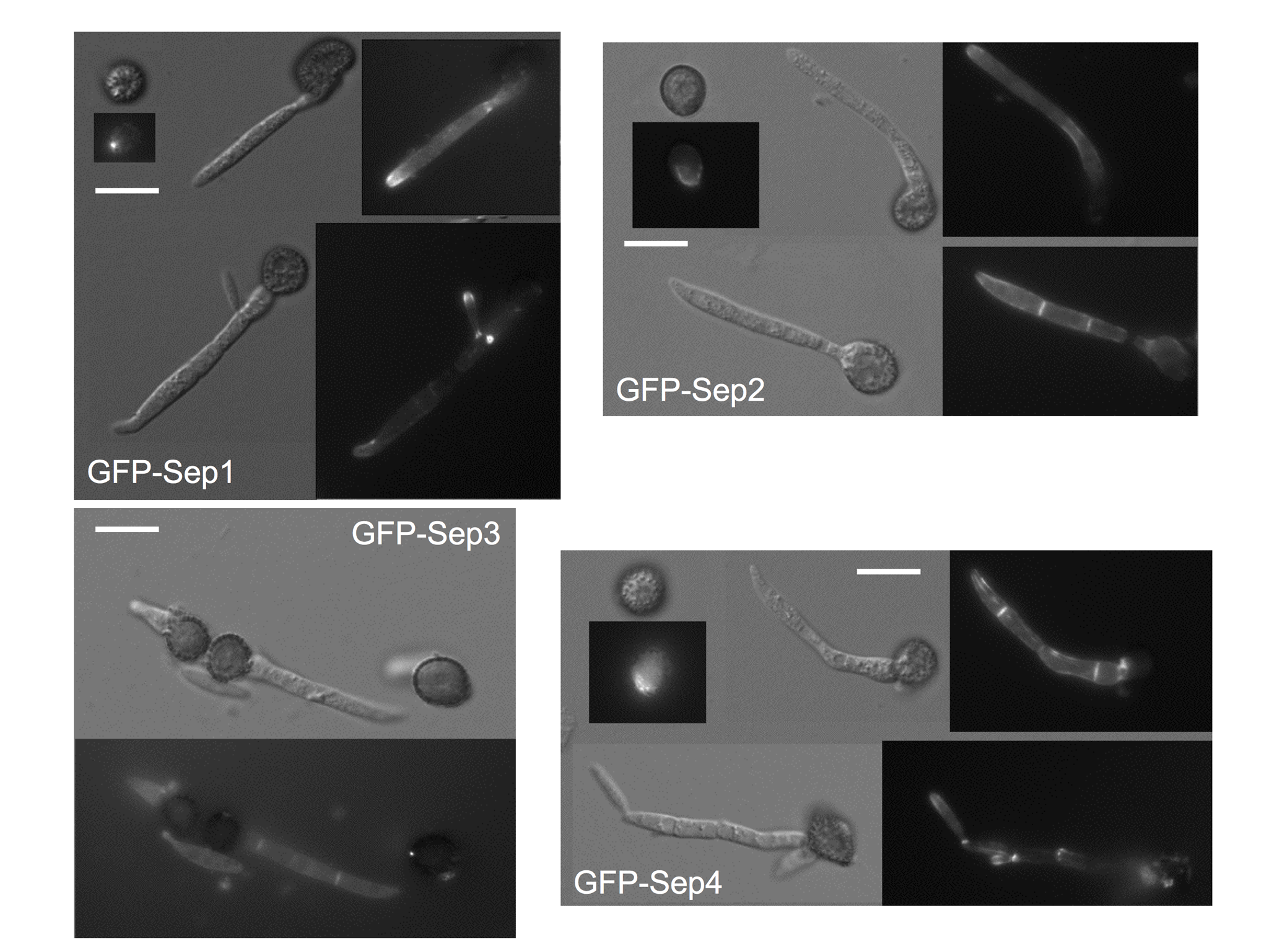

Supplement: Figure S3 — Septin localization in germinating teliospores. Corn plants were infected with crosses of compatible strains carrying the GFP-tagged septin alleles indicated. Teliospores were obtained and germinated on 2% complete medium-containing agar slides and incubated in a moist chamber at 22°C. Figures are composed images from DIC as well as epifluorescence individual images. A non-germinated teliospore can be observed as well as two distinct stages during germination process. Bar: 20 µm. (2.06 MB TIF) [file pone.0012933.s003.tif]
