# Supplementary material for: Septins from the Phytopathogenic Fungus Ustilago maydis Are Required for Proper Morphogenesis but Dispensable for Virulence
Source: PLoS One. 2010 Sep 27;5(9):e12933. doi: 10.1371/journal.pone.0012933 (PMC2946335; doi:10.1371/journal.pone.0012933)
Supplement: Table S1 — U. maydis strains used in this study. (0.09 MB DOC) [file pone.0012933.s004.doc]

**Table S1.** *U. maydis* strains used in this study

| Strain | Relevant genotype | Source |
| --- | --- | --- |
| FB1 | *a1 b1* | [47] |
| FB2 | *a2 b2* | [47] |
| UMI47 | *a1b1 GFP-sep1* | This work |
| UMI48 | *a1b1 GFP-sep2* | This work |
| UMI59 | *a1b1 GFP-sep3* | This work |
| UMI49 | *a1b1 GFP-sep4* | This work |
| UMI41 | *a1b1 sep1*∆*::hyg* | This work |
| UMI42 | *a1b1 sep2* ∆*::hyg* | This work |
| UMI68 | *a1b1 sep3* ∆*::hyg* | This work |
| UMI44 | *a1b1 sep4* ∆*::hyg* | This work |
| UMI148 | *a2b2 sep1*∆*::nat* | This work |
| UMI149 | *a2b2 sep2*∆*::nat* | This work |
| UMI150 | *a2b2 sep3*∆*::nat* | This work |
| UMI151 | *a2b2 sep4*∆*::nat* | This work |
| UMI86 | *a1b1 sep1* ∆*::hyg NLS-GFP* | This work |
| UMI87 | *a1b1 sep2* ∆*::hyg NLS-GFP* | This work |
| UMI88 | *a1b1 sep3* ∆*::hyg NLS-GFP* | This work |
| UMI89 | *a1b1 sep4* ∆*::hyg NLS-GFP* | This work |
| UMI94 | *a1b1 NLS-GFP* | This work |
| UMP61 | *a1b1 tub1-GFP* | [13] |
| UMP95 | *a1b1 fim1-GFP* | [39] |
| UMI92 | *a1b1 sep2* ∆*::hyg GFP-sep1* | This work |
| UMI193 | *a1b1 sep3* ∆*::hyg GFP-sep1* | This work |
| UMI69 | *a1b1 sep4* ∆*::hyg GFP-sep1* | This work |
| UMI91 | *a1b1 sep1* ∆*::hyg GFP-sep2* | This work |
| UMI194 | *a1b1 sep3* ∆*::hyg GFP-sep2* | This work |
| UMI76 | *a1b1 sep4* ∆*::hyg GFP-sep2* | This work |
| UMI177 | *a1b1 sep1* ∆*::hyg GFP-sep3* | This work |
| UMI178 | *a1b1 sep2* ∆*::hyg GFP-sep3* | This work |
| UMI71 | *a1b1 sep4* ∆*::hyg GFP-sep3* | This work |
| UMI77 | *a1b1 sep1* ∆*::hyg GFP-sep4* | This work |
| UMI78 | *a1b1 sep3* ∆*::hyg GFP-sep4* | This work |
| UMI79 | *a1b1 sep2* ∆*::hyg GFP-sep4* | This work |
| UMI157 | *a1b1 GFP-sep4 tub1-RFP* | This work |
| AB31 | *a2 P_crg1_:bW2* *P_crg1_:bE1* | [48] |
| UMI51 | *a2 P_crg1_:bW2* *P_crg1_:bE1 GFP-sep1* | This work |
| UMI52 | *a2 P_crg1_:bW2* *P_crg1_:bE1 GFP-sep2* | This work |
| UMI53 | *a2 P_crg1_:bW2* *P_crg1_:bE1 GFP-sep3* | This work |
| UMI54 | *a2 P_crg1_:bW2* *P_crg1_:bE1 GFP-sep4* | This work |
| UMI38 | *a2 P_crg1_:bW2* *P_crg1_:bE1 sep1*∆*::hyg* | This work |
| UMI45 | *a2 P_crg1_:bW2* *P_crg1_:bE1 sep2*∆*::hyg* | This work |
| UMI39 | *a2 P_crg1_:bW2* *P_crg1_:bE1 sep3*∆*::hyg* | This work |
| UMI40 | *a2 P_crg1_:bW2* *P_crg1_:bE1 sep4*∆*::hyg* | This work |
