# Supplementary material for: Septins from the Phytopathogenic Fungus Ustilago maydis Are Required for Proper Morphogenesis but Dispensable for Virulence
Source: PLoS One. 2010 Sep 27;5(9):e12933. doi: 10.1371/journal.pone.0012933 (PMC2946335; doi:10.1371/journal.pone.0012933)
Supplement: Table S2 — Oligonucleotide primers used in this study. (0.06 MB DOC) [file pone.0012933.s005.doc]

**Table S2.** Oligonucleotide primers used in this study

| **Name** | **Sequence 5’-3’** |
| --- | --- |
| SEP1-1 | 5’GGCACTAGTCGTGAATATGACCATGAACAA3’ |
| SEP1-2 | 5’GGTGGCCATCTAGGCCTTTGGATGTAGAGATGTGTC3’ |
| SEP1-3 | 5’ATAGGCCTGAGTGGCCTCAACTCTTTCTTGGCTTTCGAC3’ |
| SEP1-4 | 5’GGCACTAGTGTCGAATTGCGGCTGGTGCTC3’ |
| SEP1-10 | 5’GGTACCATGAACAACGAGTCGCAGCCA3’ |
| SEP1-11 | 5’GTGAGAACCTCCGTTCATATGGTCTAGATTTGGATG3’ |
| SEP1-12 | 5’CATCCAAATCTAGACCATATGAACGGAGGTTCTCAC3’ |
| SEP1-13 | 5’GGTACCTGACAAGGAGGAGAAGAAATG3’ |
| SEP2-1 | 5’GGCACTAGTGTGCAGTTGACCGTTCATAGG3’ |
| SEP2-2 | 5’GGTGGCCATCTAGGCCACGAGGTACTGATCCGGATCA3’ |
| SEP2-3 | 5’ATAGGCCTGAGTGGCCAACCAGAAGTTCGTGTATCGTAC3’ |
| SEP2-4 | 5’GGCACTAGTTGTGGTCCAGCCTTGGTGCTC3’ |
| SEP2-10 | 5’GGTACCGACAAGCCACGGGTGACTGAC3’ |
| SEP2-11 | 5’GATGCCGTTGGTAGCCATATGTGCGGCTAGTGTAGT3’ |
| SEP2-12 | 5’ACTACACTAGCCGCACATATGGCTACCAACGGCATC3’ |
| SEP2-13 | 5’GGTACCACATGCGCCACTCAAACTGGC3’ |
| SEP3-1 | 5’GGCACTAGTCCAGATGACGAATGCAGTTTT3’ |
| SEP3-2 | 5’GGTGGCCATCTAGGCCACGCGAAGCGCAGAGAGAGCC3’ |
| SEP3-3 | 5’ATAGGCCTGAGTGGCCGAATGACCTACCCGGCACGTGTT3’ |
| SEP3-4 | 5’GGCACTAGTATCGTCATCACGGGGACCCCC3’ |
| SEP3-10 | 5’GGTACCGTAGAGCTCCGTCCAGATGAC3’ |
| SEP3-11 | 5’GCGTCGACGCGCGGCCATATGGGAGTTGATGACGGA3’ |
| SEP3-12 | 5’TCCGTCATCAACTCCCATATGGCCGCGCGTCGACGC3’ |
| SEP3-13 | 5’GGTACCGGCGTACAGATGGATCTGAAC3’ |
| SEP4-1 | 5’GGCACTAGTGGTTATAGAGCGAAGCAGACT3’ |
| SEP4-2 | 5’GGTGGCCATCTAGGCCGATCTGTGCGGTGCTGAGGGT3’ |
| SEP4-3 | 5’ATAGGCCTGAGTGGCCTTTTGTTCATGTATGCACATTCT3’ |
| SEP4-4 | 5’GGCACTAGTTCCGAGTCGTGGTTGGAGCGA3’ |
| SEP4-10 | 5’GGTACCTTGCATCCTGAACGAAGTGGT3’ |
| SEP4-11 | 5’CGATAGTACCGCCGTCATATGGAACCACGATGTGAT3’ |
| SEP4-12 | 5’ATCACATCGTGGTTCCATATGACGGCGGTACTATCG3’ |
| SEP4-13 | 5’GGTACCCTGTGTGCAGATCACGAATAC3’ |
